# Coenzyme Q10 inhibits the activation of pancreatic stellate cells through PI3K/AKT/mTOR signaling pathway

**DOI:** 10.18632/oncotarget.21247

**Published:** 2017-09-23

**Authors:** Ran Xue, Jing Yang, Jing Wu, Qinghua Meng, Jianyu Hao

**Affiliations:** ^1^ Department of Gastroenterology, Beijing Chao-Yang Hospital, Capital Medical University, Beijing 100020, China; ^2^ Department of Critical Care Medicine of Liver Disease, Beijing You-An Hospital, Capital Medical University, Beijing 100069, China

**Keywords:** pancreatic stellate cell, activation, coenzyme Q10, autophagy, reactive oxygen species

## Abstract

**Aim:**

Pancreatic stellate cells (PSCs) have a vital role in pancreatic fibrosis accompanied by pancreatic ductal adenocarcinoma (PDAC) and chronic pancreatitis (CP). Any agents which can affect the activation of PSCs could become potential candidates for treatment strategies in PDAC and CP. Our aim was to explore the effect of Coenzyme Q10 (CoQ10) in the process of PSCs activation.

**Methods:**

Isolated PSCs from C57BL/6 mice were treated with various dosages of CoQ10 (1, 10, and 100μM) and different time (24h, 48h, and 72 h). Effect of CoQ10 on autophagy, apoptosis, senescence and oxidative stress, as well as the activation of PSCs were analyzed by immunocytofluorescent staining, quantitative real time RT-PCR, western blotting, SA-β-galactosidase staining, malondialdehyde and reactive oxygen species (ROS) assay.

**Results:**

Expression of α-smooth muscle actin, LC3II, Beclin1, Cleaved caspases-3 and Bax levels were significantly reduced in CoQ10 treatment groups. Meanwhile, compared with the control group, significant differences for the expression of desmin, P62, Bcl-2, p-PI3K, p-AKT and p-mTOR levels in CoQ10 treatment groups were found. Moreover, CoQ10 affected the secretion of extracellular matrix components for PSCs. Few SA-β-gal positive cells were found in CoQ10 treated groups. A significant decrease in ROS positive cells and malondialdehyde levels were observed after 72 h exposure to CoQ10.

**Conclusions:**

Our finding suggests that CoQ10 inhibits the activation of PSCs by suppressing autophagy through activating the PI3K/AKT/mTOR signaling pathway. CoQ10 may act as a therapeutic agent in PSC-relating pathologies and/or anti-fibrotic approaches.

## INTRODUCTION

In 1998, pancreatic stellate cells (PSCs) were firstly authenticated and characterized by stelliform cells in the pancreas [1]. In healthy pancreas, PSCs are inactive and accompanied by the lipid droplets that contain vitamin A. As the response of inflammation of pancreatic injury, they were awakened from their inactive phenotype into highly proliferative myofibroblast-like cells with extracellular matrix components (EMCs) expressions and α-smooth muscle actin (α-SMA) positive [2].

In the pancreas, PSCs build up only about 4–7% of the organ [1] and, in contrast to the more abundant islets or acinar cells, neither secrete hormones nor digestive enzymes. However, in pancreatic ductal adenocarcinoma (PDAC) and chronic pancreatitis (CP), it is the activated PSCs that deposit collagen fibers and contribute to the progression of pancreatic fibrosis [3–4]. Recently, activated PSCs have been the focus of multiple studies and continue to attract a lot of interest, especially related to pancreatic cancer. PSCs have not only been shown to form a dense fibrotic stroma and interact with cancer cells, but may also be capable of travelling within the body to colonize distant metastases [5–6]. Therefore, the in-depth study of the processes involved in PSCs activation is of critical importance for the development of effective therapeutic approaches for pancreas related diseases, such as PDAC and CP. Any agents which can inhibit the activation of PSCs could become potential candidates for treatment strategies in PDAC and CP.

Coenzyme Q10 (CoQ10), commonly known as ubiquinone, is a lipid-soluble, a powerful antioxidant, and an essential cofactor in mitochondrial oxidative phosphorylation [7–8]. CoQ10 has roles in many physiological processes, including sulfide oxidation, regulating the mitochondrial permeability transition pore, and the translocation of protons and Ca2+ across biological membranes [9]. Recently, many studies have shown that CoQ10 is beneficial in treating cancers that required chemotherapy and other diseases like statin myopathy, congestive heart failure and hypertension [10].

Many studies have demonstrated that oxidative stress (OS) exists in CP and PDAC proven by reduced sustains antioxidant (AO) capacity and increased levels of products of OS in patients with CP and PDAC [11–12]. Moreover, antioxidant supplementation can lead to a significant reduction in pain, and also lowered the OS in patients with idiopathic or alcoholic CP [13–14]. Besides, animal studies have shown a cessation of fibrotic cascade accompanying with antioxidant supplementation. Antioxidant treatment alleviated high glucose-induced PSC activation in primary rat PSCs [15].

CoQ10 is a strong antioxidant found in humans, however, whether CoQ10 can affect the activation of PSCs remains unclear until now. Hence, in this study, we aimed to explore the effect of CoQ10 applied to the process of PSCs activation. Our research can provide more groundwork for CoQ10 related clinical treatment strategies of pancreas disease, such as PDAC and CP.

## RESULTS

### Characterization of PSCs

**Figure 1:**
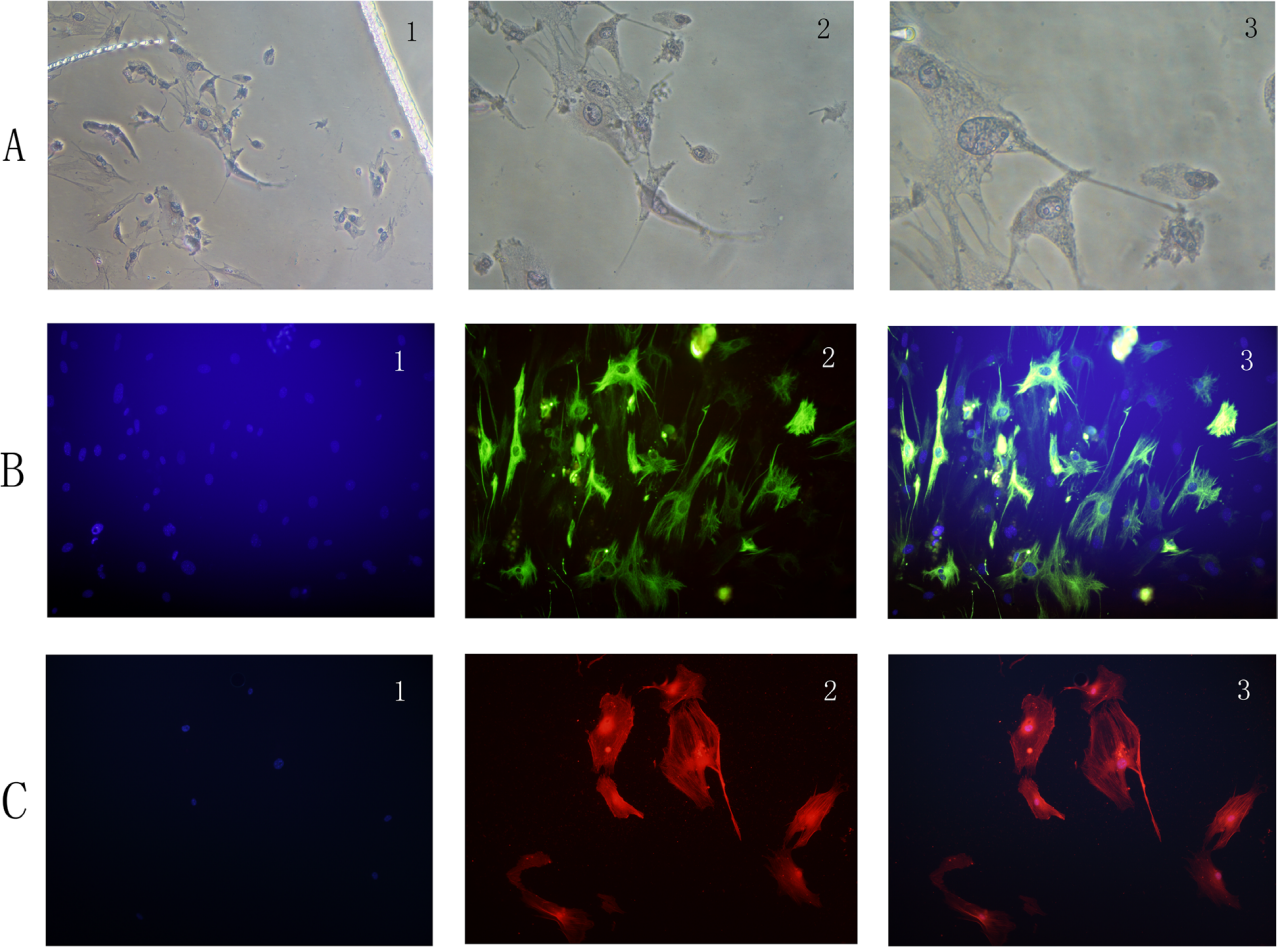
**(A)** PSCs have an angular appearance, contained lipid droplets by oil red O staining (1: original magnification, x50; 2: original magnification, x100; 3: original magnification, x200). **(B)** PSCs stain positively for desmin by IF (1: Hoechst 33258 staining, original magnification, x100; 2: Alexa Fluor 488 staining for desmin, original magnification, x100; 3: merge picture, original magnification, x100). **(C)** PSCs stain positively for α-SMA by IF (1: Hoechst 33258 staining, original magnification, x100; 2: Alexa Fluor 594 staining for α-SMA, original magnification, x100; 3: merge picture, original magnification, x100).

Approximately 4 days, the primary PSCs cells were radically crawled out, forming a “growth halo” around the tissue block. Cells had an angular appearance, contained lipid droplets by oil red O staining, and stained positively for desmin but negatively for α-SMA by IF (Figure 1A, 1B).

With passed on, cultured PSC were auto-activated, becoming myofibroblast-like cells. Cell volume of PSCs increased, and fat drops disappeared. Cells were positive for α-SMA by IF (Figure 1C).

### CoQ10 inhibited the activation of PSCs

To understand the effect of CoQ10 for the activation of PSCs *in vitro*, western blotting analysis for α-SMA, and desmin expressions of activated PSCs were performed after 24h, 48h and 72h with CoQ10 treatment.

**Figure 2:**
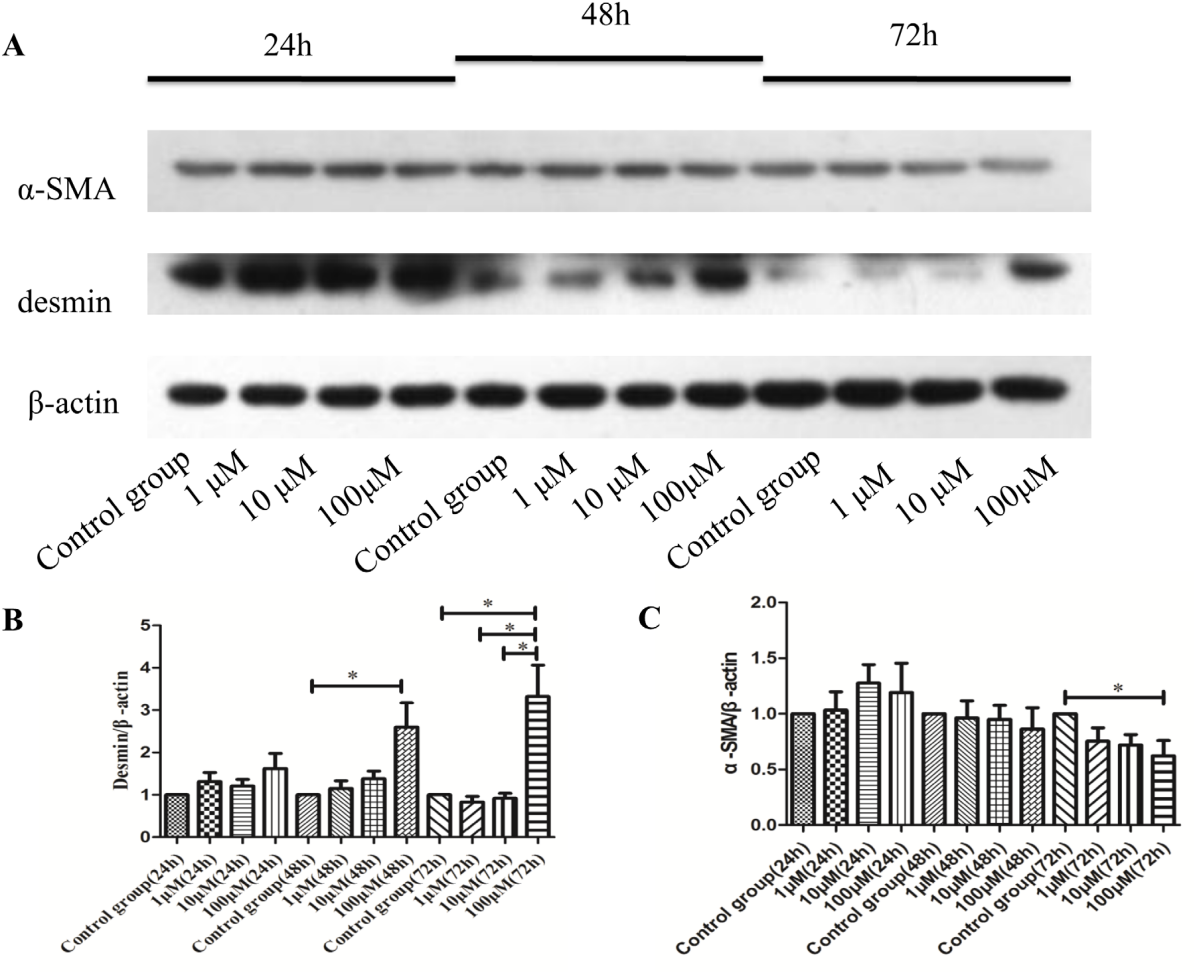
**(A)** Western blotting analysis for a-SMA, and desmin expressions of activated PSCs were performed after 24h, 48h and 72h with CoQ10 treatment. **(B)** Quantification of western blots for desmin expressions of activated PSCs after 24h, 48h and 72h with CoQ10 treatment compared to control group (^*^: P<0.05. N=3). **(C)** Quantification of western blots for a-SMA expressions of activated PSCs after 24h, 48h and 72h with CoQ10 treatment compared to control group (^*^: P<0.05. N=3).

The results showed that after 24h with CoQ10 treatment, there were no significant differences in the expression of α-SMA and desmin protein compared with control group. While, there was a significant increase of the expression of desmin protein in CoQ10 treatment groups (100μM) after 48h and 72h compared with control group (P=0.037, P=0.037). At the same time, expression of α-SMA protein was reduced in the groups which PSCs were treated with CoQ10 (100μM) after 72h compared with control group (P=0.032) (Figure 2). Because CoQ10 had a great effect on the activation of PSCs after 72 h with CoQ10 treatment; we deepen our studies on the activated PSCs after 72h with CoQ10 treatment.

### CoQ10 affected the secretion of EMCs for PSCs

**Figure 3:**
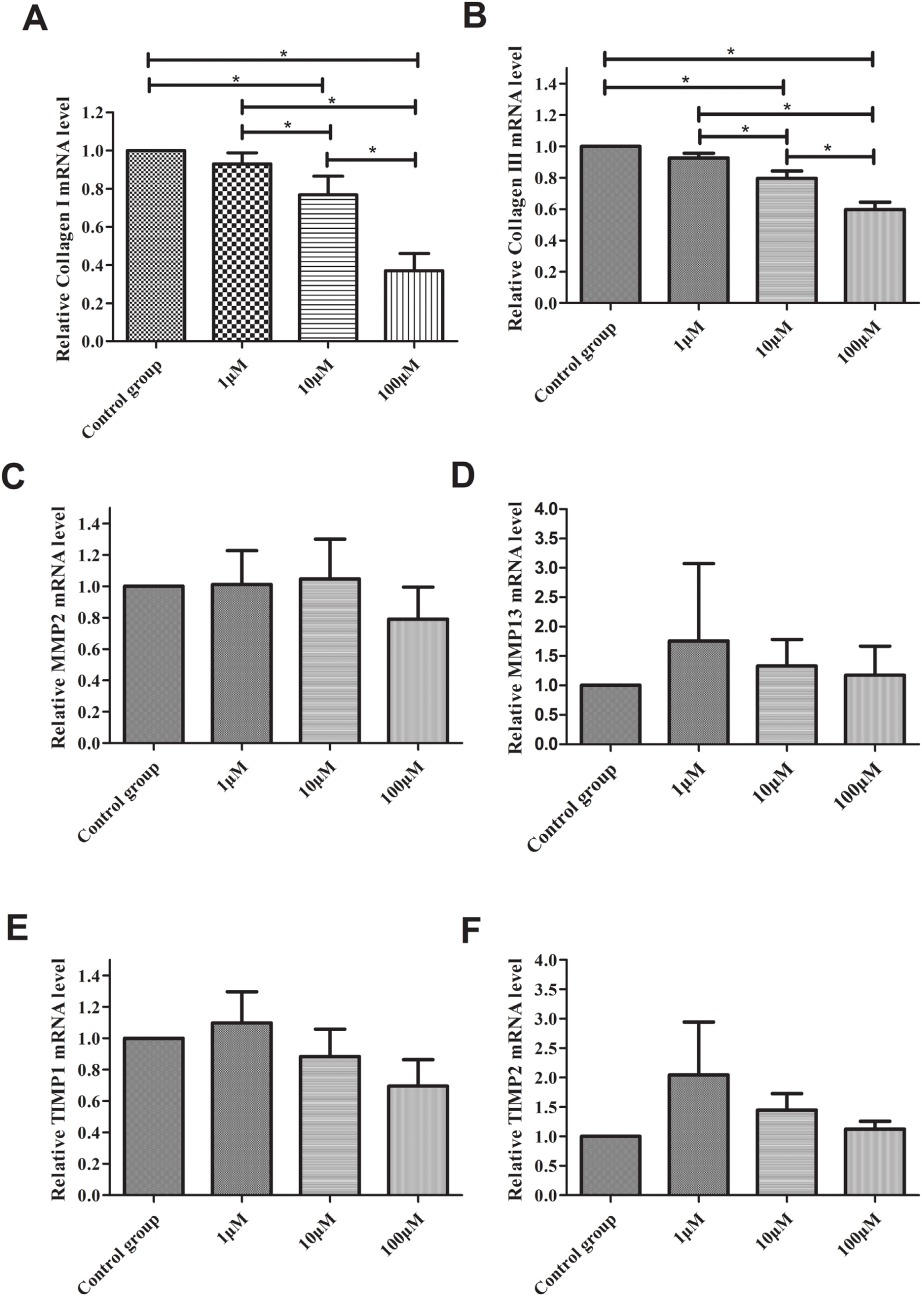
**(A)** Real-time RT-PCR analysis for Col I. **(B)** Real-time RT-PCR analysis for Col III. **(C)** Real-time RT-PCR analysis for MMP2. **(D)** Real-time RT-PCR analysis for MMP13. **(E)** Real-time RT-PCR analysis for TIMP1. **(F)** Real-time RT-PCR analysis for TIMP2. (^*^: P<0.05. N=3).

**Figure 4:**
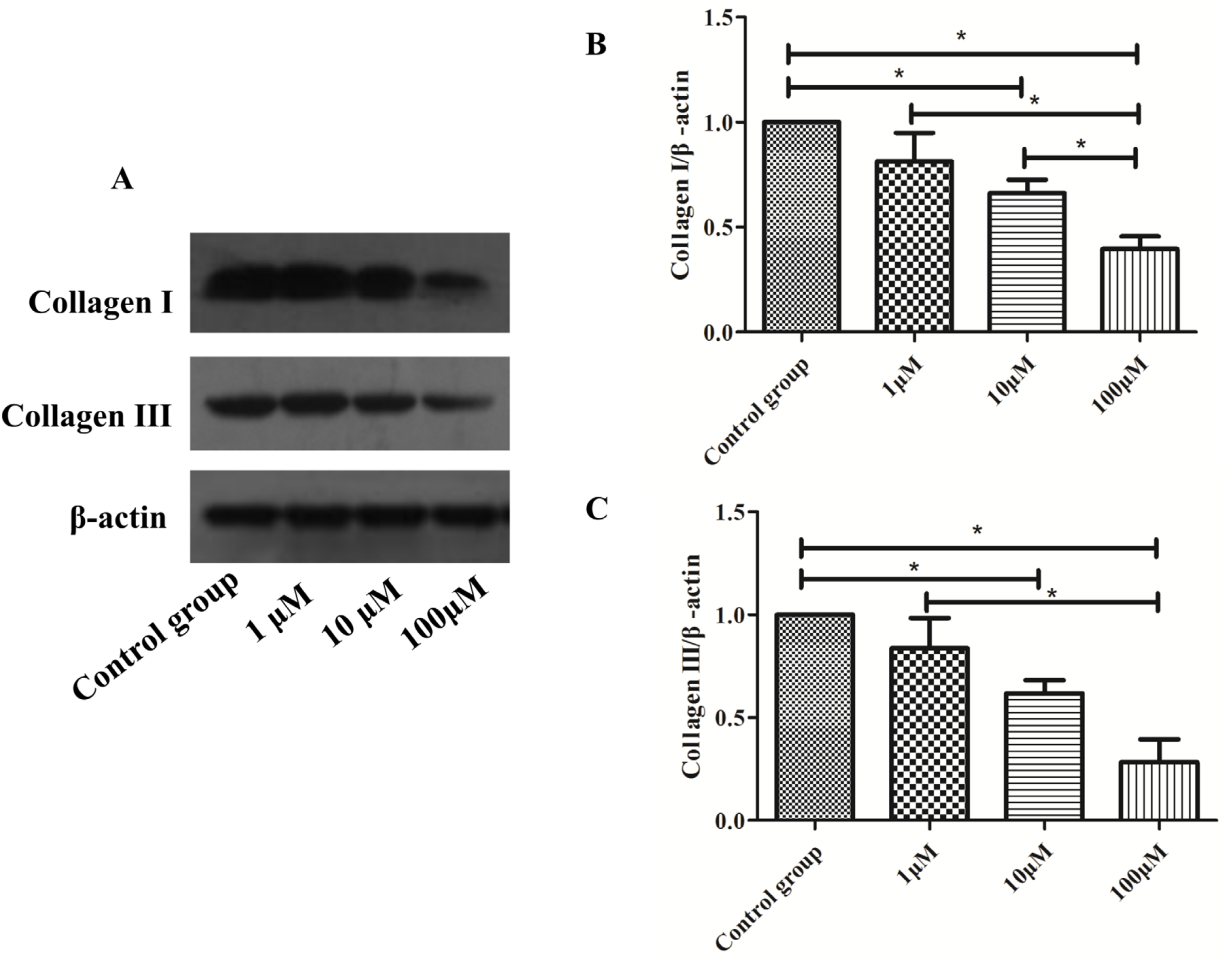
**(A)** Western blotting analysis for Col I and Col III expressions of activated PSCs were performed after 72h with CoQ10 treatment. **(B)** Quantification of western blots for Col I expressions of activated PSCs after 72h with CoQ10 treatment compared to control group (^*^: P<0.05. N=3). **(C)** Quantification of western blots for Col III expressions of activated PSCs after 72h with CoQ10 treatment compared to control group (^*^: P<0.05. N=3).

To explore the effect of CoQ10 for the secretion of EMCs for PSCs, real-time RT-PCR analysis for Collagen I (Col I), Collagen III (Col III), matrix metalloproteinase 2 (MMP2), matrix metalloproteinase 13 (MMP13), metallopeptidase inhibitor 1 (TIMP1), metallopeptidase inhibitor 2 (TIMP2) expressions of PSCs were performed. As shown in Figure 3, there were no significant differences for MMP2, MMP13, TIMP1 and TIMP2 expression between control group and CoQ10 treated groups after 72 h. Significant differences for Col I and Col III expression between control group and CoQ10 treated groups after 72 h were found (P<0.001, P<0.001). Meanwhile, there were significant decreases of Col I and Col III protein expression in CoQ10 treatment groups compared with the control group after 72h by Western blotting (Figure 4).

### Effect of CoQ10 on reactive oxygen species (ROS) production in PSCs

**Figure 5:**
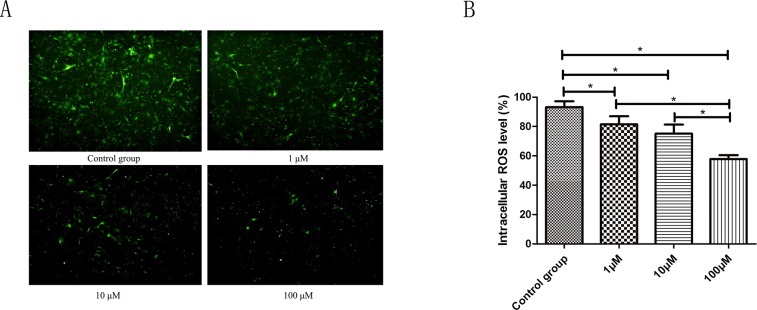
**(A)** ROS levels were tested using fluorescence microscope by the DCFH-DA fluorescent probe. A significant decrease in ROS positive cells was observed after 72 h exposure to 1.0μM, 10μM, 100μM CoQ10 as compared to the control, respectively. **(B)** ROS levels were tested using flow cytometry by the DCFH-DA fluorescent probe. There were significant differences between CoQ10 treated group and control group for ROS level of PSCs (P<0.001) (^*^: P<0.05. N=3).

ROS levels were tested using flow cytometry and fluorescence microscope by the DCFH-DA fluorescent probe. Compared to the control group, a significant decrease in ROS positive cells was observed after 72 h exposure to 1.0μM, 10μM, 100μM CoQ10, respectively. The average rate of DCF positive cells was 93.20±3.98% in the control group, while 81.46±5.52%, 75.12±6.21%, 57.81±2.69% in the 1.0 μM, 10μM, 100μM CoQ10 group contributed to ROS production (P<0.001) (Figure 5).

### Effects of CoQ10 on malondialdehyde (MDA) levels in PSCs

**Figure 6:**
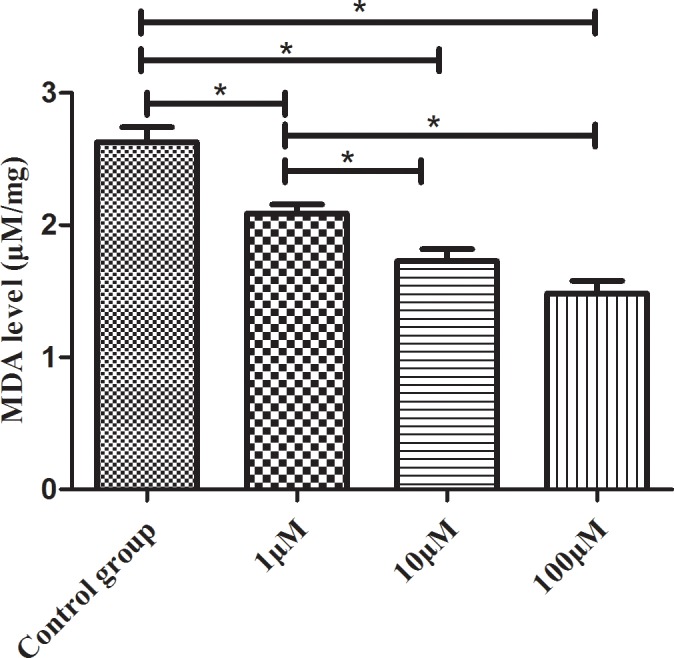
The MDA levels reduced in dose-dependent manners with CoQ10 treatment in PSCs (^*^: P<0.05. N=3)

As shown in Figure 6, the MDA levels reduced with increasing concentrations of CoQ10 (1.0, 10, 100 μM) for 72h. The PSCs exposed to 1.0, 10, 100 μM of CoQ10 had a significant lower level of MDA than that in the control group (P=0.003, P<0.001, P<0.001).

### CoQ10 suppressed cell apoptosis in the activated PSCs

In order to assess the change of cell apoptosis for activated PSCs, the expression of Cleaved Caspase-3, Caspase-3, Bax and Bcl-2 were tested by Western blotting analysis. Caspase-3 activity was also used to evaluate the level of cell apoptosis.

**Figure 7:**
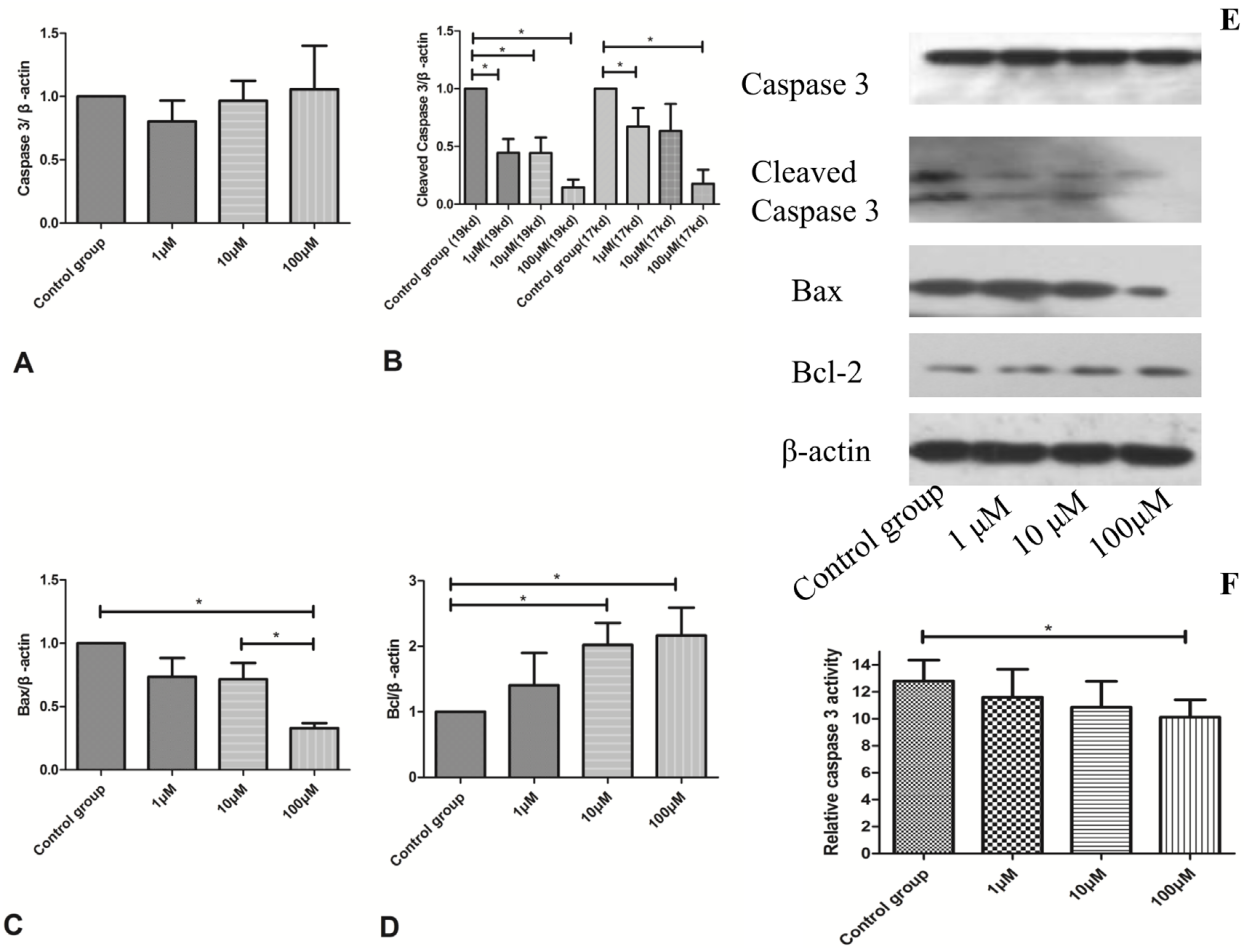
**(A)** Quantification of western blots for Caspase 3 expression of activated PSCs after 72h with CoQ10 treatment compared to control group (N=3). **(B)** Quantification of western blots for Cleaved Caspase 3 expression of activated PSCs after 72h with CoQ10 treatment compared to control group (^*^: P<0.05. N=3). **(C)** Quantification of western blots for Bax expression of activated PSCs after 72h with CoQ10 treatment compared to control group (^*^: P<0.05. N=3). **(D)** Quantification of western blots for Bcl expression of activated PSCs after 72h with CoQ10 treatment compared to control group (^*^: P<0.05. N=3). **(E)** Western blotting analysis for Caspase-3, Cleaved Caspase-3, Bcl-2 and Bax expressions of activated PSCs were performed after 72h with CoQ10 treatment. **(F)** The decreased caspase-3 activity was observed in CoQ10 treated groups compared with controls (^*^: P<0.05. N=4).

The decreased caspase-3 activity was discovered in 100μM CoQ10 treated groups compared with controls (P=0.037) (Figure 7F). The cleaved caspases-3 and Bax levels were significantly reduced in CoQ10 treated groups after 72 h (Figure 7B, 7C, 7E). In addition, CoQ10 increased Bcl-2 level of PSCs compared with the control group in a dose-dependent manner (Figure 7D, 7E).

### Effects of CoQ10 on cellular senescence

**Figure 8:**
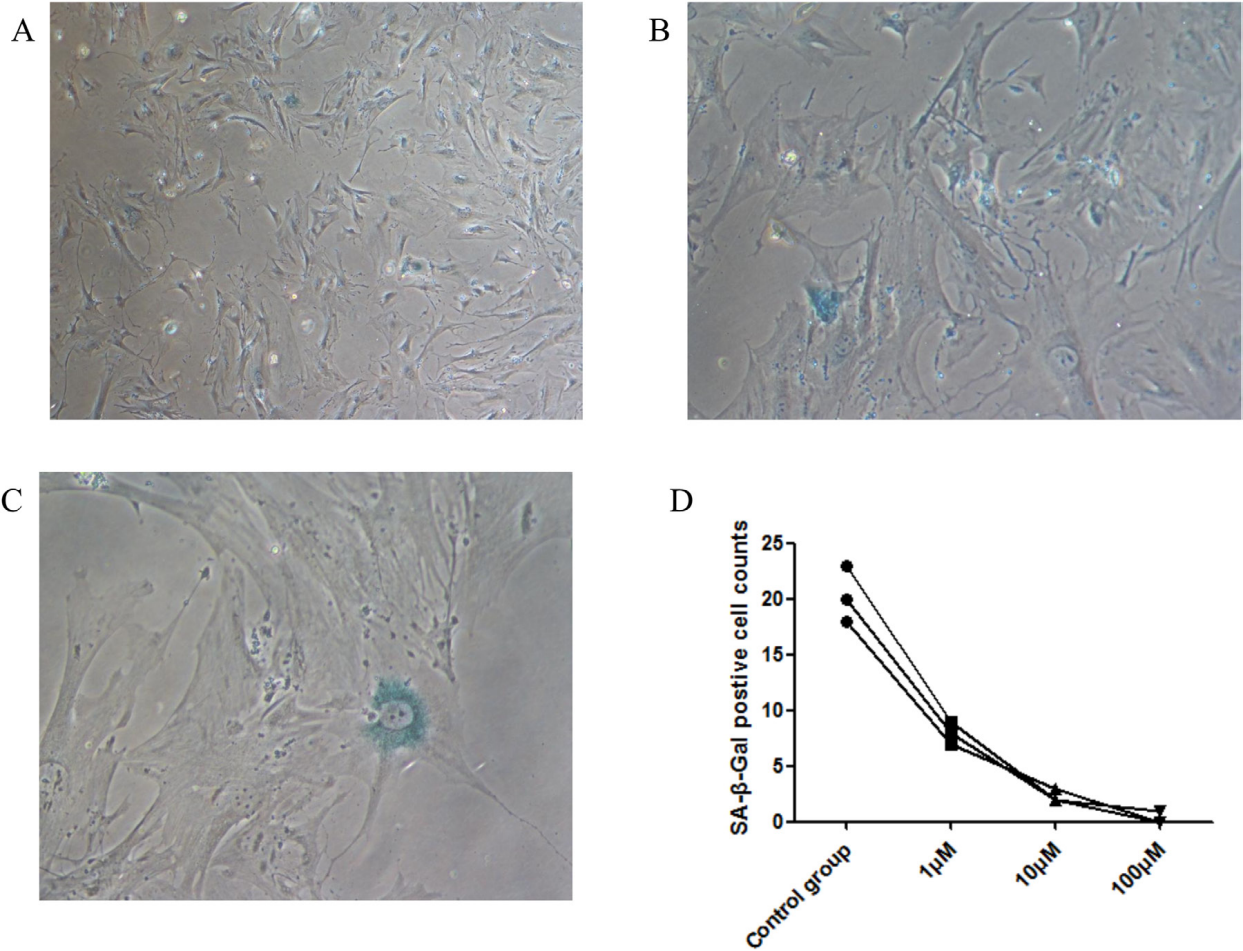
Senescence is shown in PSCs with cytoplasmic blue staining of SA-β-gal **(A)** Immunocytochemistry; original magnification, x50. **(B)** Immunocytochemistry; original magnification, x100. **(C)** Immunocytochemistry; original magnification, x200). **(D)** SA-β-gal positive cells counts decrease in dose-dependent manners with CoQ10 treatment in PSCs (N=3).

We used the SA-β-gal staining assay [16] to detect senescence in PSCs induced by CoQ10. It can be found that some cells were stained positive for SA-β-gal in PSCs control group (Figure 8). However, few SA-β-gal positive cells were found in CoQ10 treated groups. The result suggested that CoQ10 could inhibit PSC cellular senescence.

### CoQ10 suppressed cell autophagy in the activated PSCs

**Figure 9:**
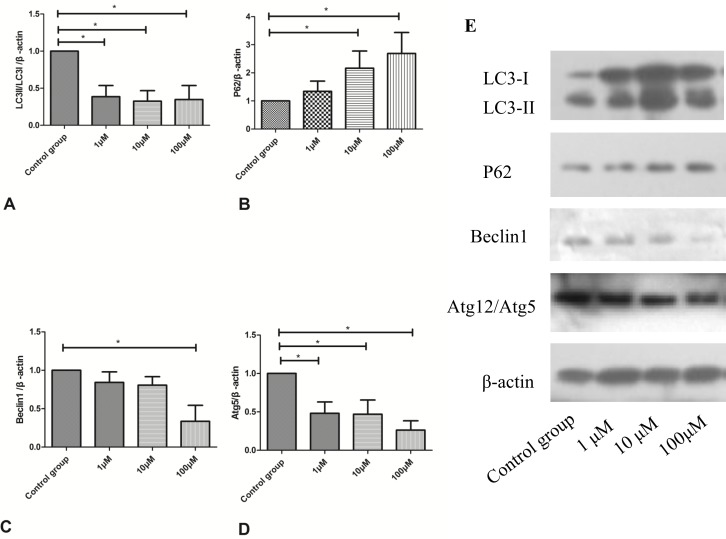
**(A)** Quantification of western blots for LC3II/LC3I expression of activated PSCs after 72h with CoQ10 treatment compared to control group (^*^: P<0.05. N=3). **(B)** Quantification of western blots for P62 expression of activated PSCs after 72h with CoQ10 treatment compared to control group (^*^: P<0.05. N=3). **(C)** Quantification of western blots for Beclin1 expression of activated PSCs after 72h with CoQ10 treatment compared to control group (^*^: P<0.05. N=3). **(D)** Quantification of western blots for Atg12/Atg5 expression of activated PSCs after 72h with CoQ10 treatment compared to control group (^*^: P<0.05. N=3). **(E)** Western blotting analysis for LC3-II, p62/SQSTM1, Beclin1 and Atg5 expressions of activated PSCs were performed after 72h with CoQ10 treatment.

As shown in Figure 9, treatment with CoQ10 reduced accumulation of LC3-II and impaired p62 clearance in both dose- and time-dependent manners. CoQ10 induced an increase in levels of p62 and a decrease in the levels of LC3-II, indicating that CoQ10 inhibited autophagy in PSCs. Additionally, CoQ10 markedly decreased levels of the early stage autophagy-related proteins Beclin1 and Atg5. All these findings suggested that CoQ10 reduced cell autophagy in the activated PSCs.

### CoQ10 inhibited cell autophagy through activating the PI3K/AKT/mTOR signaling pathway in the activated PSCs

**Figure 10:**
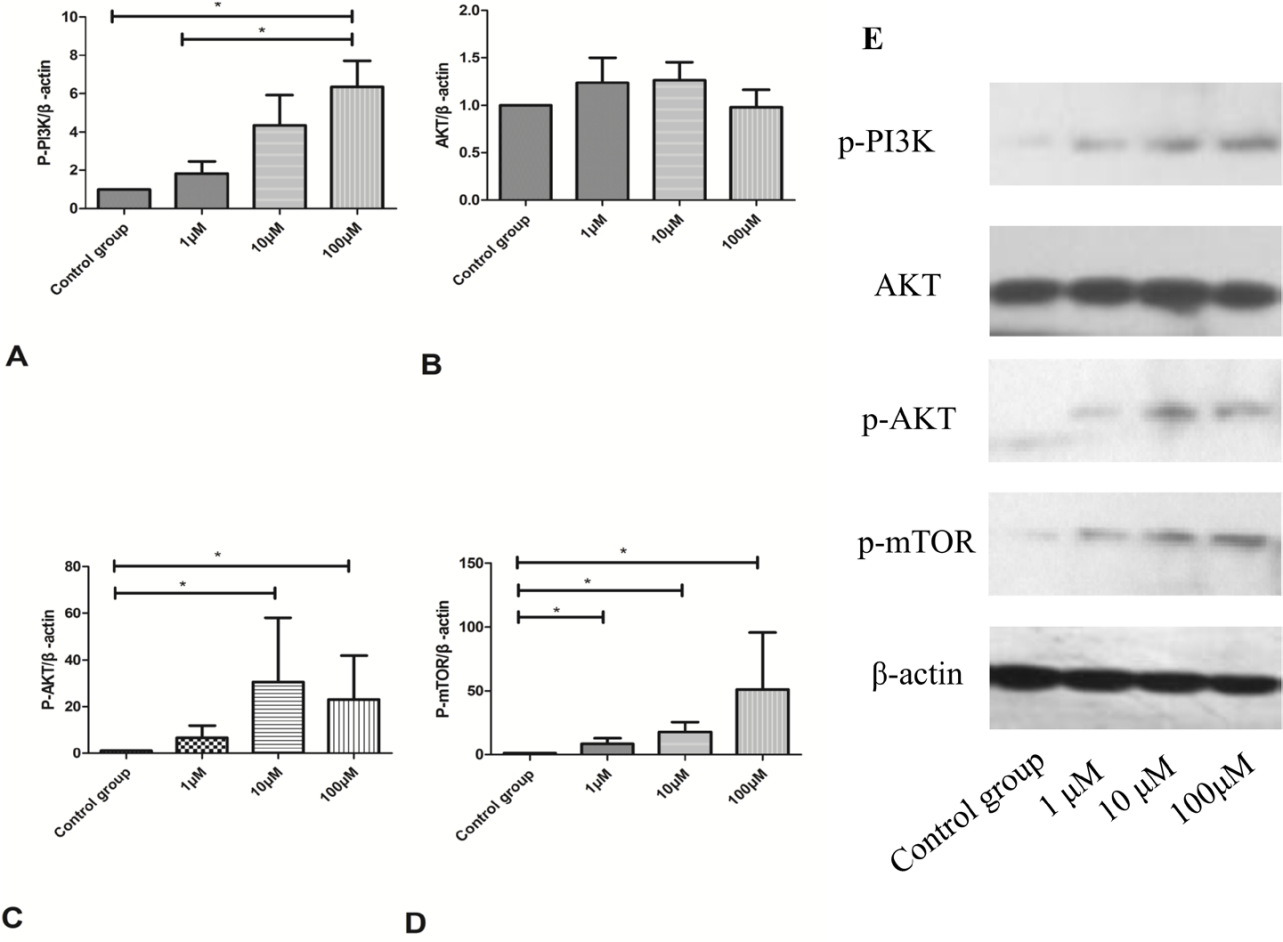
**(A)** Quantification of western blots for p-PI3K expression of activated PSCs after 72h with CoQ10 treatment compared to control group (^*^: P<0.05. N=3). **(B)** Quantification of western blots for AKT expression of activated PSCs after 72h with CoQ10 treatment compared to control group (N=3). **(C)** Quantification of western blots for p-AKT expression of activated PSCs after 72h with CoQ10 treatment compared to control group (^*^: P<0.05. N=3). **(D)** Quantification of western blots for p-mTOR expression of activated PSCs after 72h with CoQ10 treatment compared to control group (^*^: P<0.05. N=3). **(E)** Western blotting analysis for PI3K/AKT/mTOR signaling pathway proteins expression of activated PSCs were performed after 72h with CoQ10 treatment.

In Figure 10, the protein expressions of p-PI3K, p-AKT, and p-mTOR in the PI3K/AKT/mTOR signaling pathway were dose-dependently upregulated with increased CoQ10 concentrations. That is to say, CoQ10 inhibits PSCs cell autophagy through activating the PI3K/AKT/mTOR signaling pathway.

## DISCUSSION

Accumulating evidence has indicated that the activation of PSCs has a vital role during the progression of fibrogenesis in the CP and PDAC [17–18]. Therefore, the in-depth study of the processes involved in PSCs activation is of critical importance for the development of effective therapeutic approaches for pancreas related diseases, such as PDAC and CP. Any agents which can inhibit the activation of PSCs could become potential candidates for treatment strategies in PDAC and CP. This study firstly proposed that CoQ10 can inhibit the activation of PSCs by suppressing cell autophagy through the PI3K/AKT/mTOR signaling pathway. These results suggested that CoQ10 should be an effective intervention for PDAC and CP treatment. It provided some gospel for patients with PDAC and CP.

The quiescent PSCs have a lower synthesizing capacity of EMCs and hold many retinoid-containing droplets [19]. As the response of inflammation or injury events, PSCs can shift into the activated state, which can be identified with myofibrolast-like phenotype and the presence of α-SMA, as well as various kinds of growth factors and cytokines and numerous EMC proteins [20]. Under a continuous inflammatory stimulation, the activation of PSCs was substantial and consequently resulted in the development of pancreatic fibrosis [21]. Finally, the dynamic fibrotic process leads to cancer progression and anatomical anomalies [22]. So, the activation of PSCs plays a crucial role in the development of reasonable treatment strategies for CP and PDAC. The suppression of the activation of PSCs is the key to an effective intervention for CP and PDAC. In our study, CoQ10 significantly suppressed the activation of PSCs by inhibiting the production of fibrogenic mediators (such as α-SMA) and EMC proteins. It is indicated CoQ10 should be an effective approach for pancreatic fibrosis.

Autophagy is a process necessary to maintain homeostasis, especially during metabolic stress [23–24]. It has been reported that PSCs activation is related to autophagy and can promote pancreatic cancer growth and metastasis by tumor stromal interactions. Chemical and genetic autophagy inhibition lead PSCs to a quiescent state, demonstrating that autophagy inhibition in PSCs is a promising therapy for CP and PDAC [25–26]. In our study, CoQ10 was associated with the down-regulation of cell autophagy through activating the PI3K/AKT/mTOR signaling pathways. These could be the underlying mechanism for CoQ10 inhibiting the activation of PSCs.

It is widely recognized that ROS generation precedes downstream cellular cascades, including those that determine cell fate either survival (autophagy) or death (apoptosis, necrosis) [27]. ROS and programmed cell death (such as autophagy, apoptosis) can regulate each other depending on the stimulus. There exists much complexity in dissecting the interplay around ROS, apoptosis and autophagy in cell fate. Excess of ROS can cause cell death through oxidation of membrane lipids and proteins [28–30].

In this study, CoQ10 significantly reduced the intracellular level of ROS in PSCs. Because ROS can trigger autophagy and apoptosis, we suspect that CoQ10 reducing the intracellular level of ROS is the upstream event for the change of cell autophagy and apoptosis in PSCs. In addition, ROS can oxidize proteins and lipids, as well as ROS mediated fibronectin and collagen IV expression [31], which can explain the change of MDA generation after CoQ10 treatment in PSCs.

Cellular senescence can cause a state of nonreversible growth arrest, which is the result from a wide variety of stresses. Excessive productions of ROS lead to the implementation of oncogene-induced senescence, p16INK4A-induced senescence, as well as replicative senescence [32–33]. Our result suggested that CoQ10 could inhibit PSCs cellular senescence and the down-regulation of intracellular ROS in PSCs may be the underlying mechanism.

This study has some limitations. Further studies involved the *in vivo* suppressive effect of CoQ10 for the activated PSCs are still required, although cultures of PSC are considered as an appropriate *in vitro* model for the screening of anti-fibrotic agents. Collectively, CoQ10 has potent anti-fibrotic activities because of its effective suppression of the PSC-enhancing substances and gene expressions. Our study suggests that CoQ10 can act as a potential method for clinical treatment of PSC-relating pathologies, such as CP and PDAC.

## MATERIALS AND METHODS

### Animals

Healthy male C57BL/6 mice weighing 13 to 25 g and aged 3 to 10 weeks were used in our tests. They were raised in the medical research center of Beijing Chao-yang Hospital, Capital Medical University. All procedures and methods were performed according to the guidelines of the Animals Committee. The investigation has been conducted in accordance with the ethical standards and according to the Declaration of Helsinki and has been approved by Beijing Chao-yang Hospital, Capital Medical University.

### Isolation and culture of mouse PSCs

C57BL/6 mouse PSCs were primary isolated and cultured using a published method [34]. In detail, pancreatic tissues in C57BL/6 mice were collected, washed with fetal bovine serum, minced into 0.5-1 mm^3^, and cultured in sterile culture flasks. When they reached 70%-80% confluence after 4 days, primary PSCs were collected and passed on. Primary mouse PSCs were used in the second to fifth passage of this experiment.

### Identification of isolated mouse PSCs

The changes in intracellular lipid droplets were detected by oil red O staining (Sigma-Aldrich); α-SMA and desmin expression were identified via immunocytofluorescent staining and western blotting.

### Immunocytofluorescent staining

PSCs were seeded in 12-well plates (2×10^5^/well). Cells were 4% paraformaldehydefixed, immersed in 0.1% Triton X-100 for 10 min. Rabbit polyclonal anti-α-SMA (ab5694, Abcam, USA) and anti-desmin (ab32362, Abcam, USA) were incubated for 16 h at 4°C. Alexa Fluor 488-labeled Goat Anti-Rabbit IgG (H+L) (1/100) and Alexa Fluor 594-labeled Goat Anti-Rabbit IgG (H+L) (1/100) were secondary antibodies for desmin and α-SMA, respectively. Hoechst33258 (C1018, Beyotime Biotechnology, China) was also used to stain PSCs. Observation of cells was proceeded through a fluorescence microscope (LSM 510 META, Zeiss Inc., Jena, Germany). The filters used to generate the Figure 1 are shown in the Supplementary Table 1.

### CoQ10 treatment of cell lines

In every individual experiment, PSCs in the logarithmic phase was seeded at 2×10^5^cells/ml, and CoQ10 (in FBS) was added to cultures for 24h, 48h, 72 h at a final concentration of 1μM, 10μM, 100μM. Then PSCs treated with CoQ10 were collected for analysis.

### Quantitative real time RT-PCR

Guided by the manufacturer's instructions, RNeasy Mini Kit (QIAGEN, Germany) was used to isolate total RNA from PSCs cells. RT-PCR assay was performed as described previously [35]. To be brief, reverse transcription was carried with SuperScript III reverse transcriptase (Life Technologies) and 1 mg of total RNA. Gene specific primer and ampliTaq Gold 360MasterMix (Life Technologies) were used for amplification of target genes. The β-actin gene was considered as an internal standard. The list of primers was shown in Supplementary Table 2.

### Western blotting analysis

Western blotting analysis was performed as we described above [36]. Protein concentrations were measured by the bicinchoninic acid (BCA) Protein Assay kit (P0010, Beyotime Biotechnology, China). Samples of 16μg total protein were used for western blots. The list of primary antibodies was shown in Supplementary Table 3. HRP-labeled goat anti-rabbit IgG (H+L) (A0208, Beyotime Biotechnology, China) was used as the secondary antibody. Protein bands were visualized by Super Signal West Pico Chemiluminescent Substrate (Thermo Fisher Scientific, Waltham, MA, USA). The average intensities of each standard protein band were quantified using Photoshop CS5 (Adobe Systems Incorporated) and these results were normalized using β-actin. The results were column-plotted using GraphPad Prism 7 software.

### Quantification of ß-galactosidase

PSCs were seeded in a 6-well plate with 1× 10^3^ cells/cm^2^. After 3 days of culture, PSCs were rinsed with PBS and 1 ml of fixative per well was added for 20 min. Then 1 ml per well of working solution of β-galactosidase with X-Gal was placed and maintained at 37°C overnight (C0602, Beyotime Biotechnology, China). The senescent cells were observed under microscope and counted from 5 random fields of vision.

### Measurement of ROS generation

Changes in intracellular ROS levels were measured through the oxidative conversion of cell permeable 2′, 7′-dichlorofluorescein diacetate (DCFH-DA) to fluorescent di-chlorofluorescein (DCF). PSCs in 6-well culture dishes were incubated with control media or CoQ10 treated media. DCF fluorescence was detected by FACS can flow cytometer (Becton Dickinson) and fluorescence microscope (LSM 510 META, Zeiss Inc., Jena, Germany).

### Malondialdehyde (MDA) assay

For the MDA assay, proteins were prepared in the Lipid Peroxidation MDA assay kit (S0131, Beyotime Biotechnology, China). The MDA concentration of respective sample was tested by an enzyme-linked immunosorbent assay (ELISA) reader (BioTeck, USA) at 532 nm, using 490 nm as a control.

### Caspase-3 activity measurement

The activity of caspase-3 was judged by the caspase-3Activity Assay Kit (C1115, Beyotime, China). The absorbance (A405) was evaluated by an ELISA reader (BioTeck, USA) and converted to the amounts of pNA that were produced in the PSCs.

### Statistical analysis

All statistical analyses were run by SPSS 16.0 for Windows (SPSS Inc., IL, USA). Data are presented as means ± SD. The P value of < 0.05 was considered statistically significant. One-way ANOVA, Mann-Whitney U test or a two-tailed Student's t-test was used to contrast inter-group variance.

## SUPPLEMENTARY MATERIALS TABLES


