# Supplementary material for: Coenzyme Q10 inhibits the activation of pancreatic stellate cells through PI3K/AKT/mTOR signaling pathway
Source: Oncotarget. 2017 Sep 23;8(54):92300–11. doi: 10.18632/oncotarget.21247 (PMC5696182; doi:10.18632/oncotarget.21247)
Supplement: Supplementary file 1 [file oncotarget-08-92300-s001.pdf]

Coenzyme Q10 inhibits the activation of pancreatic stellate cells through PI3K/AKT/mTOR signaling pathway

SUPPLEMENTARY MATERIALS

Supplementary Table 1: The filters for Figure 1 generation

Fluorophore	Excitation peak (nm)	Emission peak (nm)
Alexa Fluor 488	488	519
Hoechst 33258	346	460
Alexa Fluor 594	591	614

Supplementary Table 2: 6 sets of primers were designed using the Primer Explorer version 4 software (Eiken Chemical Co., Ltd., Tokyo, Japan; http://primerexplorer.jp/elamp4.0.0/index.html) and synthesized by Shanghai Sangon Co., Ltd

Primer name	Length (bp)	Primer sequence(5′ to 3′)
Collagen I	26	GGGGCAAGACAGTCATCGAA
Collagen I	26	GAACCAGATTGGGGTGGAGG
Collagen III	26	GCGAGCGGCTGAGTTTTATG
Collagen III	26	GCAGCTCAGAGTAGCACCAT
MMP-13	26	ATGGTCCAGGCGATGAAGAC
MMP-13	26	GCATCAAGGGATAGGGCTGG
TIMP-1	27	CAACTCGGACCTGGATGCTAA
TIMP-1	26	ACTCTTCACTGCGGTTCTGG
MMP-2	26	AACGGTCGGGAATACAGCAG
MMP-2	26	AAACAAGGCTTCATGGGGGC
TIMP-2	26	TCTGGATGGACTGGGTCACA
TIMP-2	27	GTTGATGCAGGCGAAGAACTT

Sequences of the primers used in this study

Supplementary Table 3: Antibodies and conditions used for western blotting analyses

Antibody	Number	Species	Dilution	Source
α-SMA	ab5694	Rabbit polyclonal antibody	1:500	Abcam
desmin	ab32362	Rabbit monoclonal antibody	1:1000	Abcam
Beclin1	#3738	Rabbit polyclonal antibody	1:1000	Cell Signaling Technology
Atg5	#12994	Rabbit monoclonal antibody	1:1000	Cell Signaling Technology
LC3B	L7543	Rabbit polyclonal antibody	1:1000	Sigma
SQSTM1/p62	#5114	Rabbit polyclonal antibody	1:1000	Cell Signaling Technology
β-Actin (13E5)	#4970	Rabbit monoclonal antibody	1:1000	Cell Signaling Technology
Phospho-mTOR (Ser2448)	#2971	Rabbit polyclonal antibody	1:1000	Cell Signaling Technology
Phospho-Akt (Ser473)	#4060	Rabbit monoclonal antibody	1:1000	Cell Signaling Technology
PI3 Kinase p85 (19H8)	#4257	Rabbit monoclonal antibody	1:1000	Cell Signaling Technology
Akt	#9272	Rabbit polyclonal antibody	1:1000	Cell Signaling Technology
Bax	#2772	Rabbit polyclonal antibody	1:1000	Cell Signaling Technology
Bcl-2 (D17C4)	# 3498	Rabbit monoclonal antibody	1:1000	Cell Signaling Technology
Caspase-3 (8G10)	#9665	Rabbit monoclonal antibody	1:1000	Cell Signaling Technology
Cleaved Caspase-3 (Asp175)	#9664	Rabbit monoclonal antibody	1:1000	Cell Signaling Technology
Collagen III	ab7778	Rabbit polyclonal antibody	1:1500	Abcam
Collagen I	ab6803	Mouse monoclonal antibody	1:1000	Abcam
